# Targeting the p90RSK/MDM2/p53 Pathway Is Effective in Blocking Tumors with Oncogenic Up-Regulation of the MAPK Pathway Such as Melanoma and Lung Cancer

**DOI:** 10.3390/cells13181546

**Published:** 2024-09-14

**Authors:** Immacolata Maietta, Eleonora Viscusi, Stefano Laudati, Giuseppe Iannaci, Antonio D’Antonio, Rosa Marina Melillo, Maria Letizia Motti, Valentina De Falco

**Affiliations:** 1 Institute of Endocrinology and Experimental Oncology (IEOS), National Research Council (CNR), Via S. Pansini 5, 80131 Naples, Italy; immacolata.maietta@uvigo.gal (I.M.); rosmelil@unina.it (R.M.M.); 2 U.O.C. Anatomia Patologica, P.O. Pellegrini ASL NA1 Centro, 80134 Naples, Italy; eleonora.viscusi@libero.it (E.V.); giuseppe.iannaci@aslnapoli1centro.it (G.I.); 3 U.O.C. Anatomia Patologica, Ospedale del Mare ASL NA1 Centro, 80147 Naples, Italy; stefano.laudati@aslnapoli1centro.it (S.L.); antonio.dantonio@aslnapoli1centro.it (A.D.); 4 Department of Molecular Medicine and Medical Biotechnology, University of Naples Federico II, 80131 Naples, Italy; 5 Department of Medical, Movement and Wellbeing Sciences, University of Naples Parthenope, 80133 Naples, Italy

**Keywords:** p90RSK, p53, MDM2, apoptosis, tumorigenesis, cell proliferation, cancer, targeted therapy, cancer drug resistance

## Abstract

In most human tumors, the MAPK pathway is constitutively activated. Since p90RSK is downstream of MAPK, it is often hyperactive and capable of phosphorylating oncogenic substrates. We have previously shown that p90RSK phosphorylates MDM2 at S166, promoting p53 degradation in follicular thyroid carcinomas. Thus, the inhibition of p90RSK restores p53 expression, which in turn inhibits cell proliferation and promotes apoptosis. In the present study, we demonstrated that the p90RSK/MDM2/p53 pathway proved to be an excellent target in the therapy of tumors with MAPK hyperactivation. For this purpose, we selected p53wt melanoma, lung and medullary thyroid carcinoma cell lines with high activation of p90RSK. In these cell lines, we demonstrated that the p90RSK/MDM2/p53 pathway is implicated in the regulation of the cell cycle and apoptosis through p53-dependent transcriptional control of *p21* and *Bcl-2*. Furthermore, with an immunohistochemical evaluation of primary melanomas and lung tumors, which exhibit highly activated p90RSK compared to corresponding normal tissue, we demonstrated that MDM2 stabilization was associated with p90RSK phosphorylation. The results indicate that p90RSK is able to control the proliferative rate and induction of apoptosis through the regulation of p53wt levels by stabilizing MDM2 in selected tumors with constitutively activated MAPKs, making p90RSK a new attractive target for anticancer therapy.

## 1. Introduction

The mitogen-activated protein kinase (MAPK) pathway is involved in the regulation of very important key cellular processes. Extracellular stimuli such as hormones, growth factors and neurotransmitters induce the RAS/MAPK pathway through the activation of a cellular receptor, which, in turn, by activating the small GTPases of the RAS family, transfers the signal to RAF, MEK1/2 and ERK1/2 [1]. Once activated, ERK1/2 phosphorylate several substrates in cells regulating cell proliferation, survival, metabolism, migration and differentiation [1].

This signal transduction pathway is overactivated in many human tumors [2].

Over 85% of human tumors present a hyperactivated MAPK pathway due to oncogenic activating mutations in its components, including RTK, RAS and BRAF [3,4,5], which significantly promote disease progression [6].

Several inhibitors targeting KRAS, BRAF and MEK are commonly used in clinical practice. However, the development of drug resistance mechanisms in patients who initially responded well to therapy significantly limits the effectiveness of these inhibitors, making it necessary to identify new drugs capable of overcoming resistance mechanisms but above all new molecular targets [7,8,9,10].

Unlike RAS and RAF, MEK and ERK mutations occur rarely and sometimes cause resistance to RAF inhibitors (RAFis) [8,11,12,13].

Among the most downstream substrates of MAPKs is the serine–threonine kinase p90RSK [12].

The 90-kDa S6 ribosomal kinase (p90RSK) family is composed of four highly similar Ser/Thr kinases (RSK1-4) that act downstream of the RAS/mitogen-activated protein kinase (MAPK) signaling pathway. p90RSKs have an N-terminal kinase domain (NTKD) and a C-terminal kinase domain (CTKD). The effector domain of the kinase is the NTKD, and because it phosphorylates several substrates that contain a basophilic consensus region (Arg-Arg-X-Ser/Thr or Arg/Lys-X-Arg-X-X-Ser/Thr) [14,15], it belongs to the AGC kinases (such as PKA, PKG and PKC). CTKD has a regulatory function and it is a calcium/calmodulin-dependent protein kinase; a linker region separates these domains. Finally, an ERK1/2 docking site called the D domain is located in the C-terminal region [16,17].

For the full activation of p90RSK, many of its residues, including Ser221, Ser380, Ser363, Thr359 and Thr573 (numbers refer to RSK1 isoform) [18], must be phosphorylated first by ERK and phosphoinositide-dependent kinase 1 (PDK1) and subsequently through the autophosphorylation of the N-terminal kinase domain (NTKD) by the C-terminal kinase domain (CTKD) [14,19].

However, although these proteins are functionally partially redundant, particular cellular processes are regulated by some specific isoforms since each is preferentially expressed in some tissues and phosphorylates isoform-specific substrates [20,21,22].

The RSK1 and/or RSK2 isoforms are overexpressed in many tumors, including breast and prostate carcinomas and osteosarcoma [23,24,25]; these isoforms promote tumor growth, survival, invasion and metastasis [25,26], and for this reason, they constitute promising anti-tumor targets.

In contrast, the role of RSK3 and RSK4 is not fully defined; they have been described as tumor suppressors, and their down-regulation in tumors predicts a poor prognosis. They are also involved in resistance to inhibitors of PI3K in breast cancer [27]; moreover, in renal cell carcinoma and melanoma cell lines, it has been demonstrated that the RSK4 isoform plays a role in resistance to sunitinib [28].

Since the RAS/mitogen-activated protein kinase (MAPK) pathway regulates multiple important biological processes such as cell proliferation, migration and survival, and p90RSK is overactivated in many human tumors such as lung cancer, breast cancer and prostate cancer, in recent years, the possibility has emerged of considering p90RSK an excellent target in anti-tumor therapy. In this context, it has been suggested that p90RSK has a significant function in generating resistance to KRAS/BRAF/MEK inhibitors [8,9,10]. However, effective p90RSK inhibitors are not yet available in the clinic [29].

Starting from the mechanism demonstrated in our previous work in follicular thyroid tumors, in which the inhibition of p90RSK triggers a decrease in cell proliferation and the activation of apoptosis through the MDM2/p53 pathway, here, we demonstrate that the inhibition of the p90RSK/MDM2/p53 pathway is critical in inducing a blockade of proliferation and apoptosis by regulating p53wt levels through the stabilization of MDM2 in selected tumors with constitutively activated MAPKs such as melanoma and lung cancer. This does not occur in prostate cancer, where p90RSK appears less active.

This described mechanism makes the idea of considering p90RSK an excellent target for anti-tumor therapy in tumors with an activated MAPK pathway even more intriguing.

## 2. Materials and Methods

### 2.1. Cell Lines

The human melanoma cell line A375 was purchased from the ATCC (Manassas, VA, USA). The culture medium used for A375 melanoma cell line was RPMI-1640 supplemented with 10% fetal bovine serum (FBS), and cells were maintained in 100 mm tissue culture dishes at 37 °C in humidified air with 5% CO_2_. A375 cells harbor BRAF (p Val600Glu) and CDKN2A (p.Glu61Ter) homozygous mutations and a TERT (c.1-146C > T) mutation.

The human lung carcinoma cell line A549 was obtained from the ATCC (Manassas, VA, USA). A549 cells derived from a non-small cell epithelial lung adenocarcinoma were grown in DMEM supplemented with 10% fetal bovine serum (FBS) and maintained in 100 mm tissue culture dishes at 37 °C in humidified air with 5% CO_2_. A549 cells harbor KRAS (p.Gly12Ser) and STK11 (p.Gln37Ter) homozygous mutations.

The human medullary thyroid carcinoma cell line MZ-CRC-1 was kindly provided by Robert F. Gagel. The MZ-CRC-1 cells, derived from a malignant pleural effusion from a patient with a metastatic MTC [30], were grown in DMEM supplemented with 10% FBS and maintained in 100 mm tissue culture dishes at 37 °C in humidified air with 5% CO_2_. MZ-CRC-1 cells harbor a heterozygous (ATG to ACG) transition in *RET* exon 16, resulting in the MEN2B-associated substitution of threonine 918 for methionine (M918T) [31].

The human epithelial prostatic carcinoma cell line LNCaP was purchased from the ATCC (Manassas, VA, USA). The culture medium used for LNCaP cells was RPMI-1640 supplemented with 10% FBS, and cells were maintained in 100 mm tissue culture dishes at 37 °C in humidified air with 5% CO_2_. LNCaP cells derive from lymph node carcinoma of the prostate and harbor PTEN (p.Lys6Argfs*4), AR (p.Thr878Ala), MEN1 (p.Tyr318Ter) and PIK3R1 (p.Arg639Ter) mutations.

All media were supplemented with 100 U/mL penicillin–streptomycin and 2 mM L-glutamine (GIBCO, Paisley, PA, USA).

Cell lines were checked monthly to confirm the absence of mycoplasma contamination.

The information on mutations was collected from https://web.expasy.org/cellosaurus (accessed on 25 March 2024).

### 2.2. Compounds

BI-D1870 powder (Selleckchem, Houston, TX, USA) was dissolved with DMSO to a 10 mM stock solution and stored at −80 °C.

Wortmannin powder (Cell Signaling Technologies, Danvers, MA, USA) was dissolved with DMSO to a 2 mM stock solution and stored at −20 °C.

### 2.3. Protein Extraction, Western Blotting and Antibodies

Cells were lysed in JS buffer (50 mM Hepes, pH 7.5, 150 mM NaCl, 1% glycerol, 1% Triton X-100, 5 mM EGTA, 1.5 mM MgCl2, 50 mM NaF, 10 mM Na_3_VO_4_, 28 µg of aprotinin/mL, 10 µg of leupeptin/mL, 1 mM PMSF) and centrifugated at 10,000× *g*. The concentration of protein was quantified using a Bradford assay (Bio-Rad Laboratories, Berkeley, CA, USA). Antigens were highlighted using an ECL kit assay (Amersham Pharmacia Biotech, Amersham, UK). Total lysates were separated on acrylamide gel at different concentrations (SDS–PAGE). To perform immunoblotting, proteins were transferred onto nitrocellulose filters (PerkinElmer, Waltham, MA, USA). First, the membranes were blocked with a solution of 5% BSA, 1× TBS and 0.1% Tween-20 for 1 h, and then the membranes were hybridized with the different antibodies. Anti-phospho-S166-MDM2 (#3521), anti-RSK1/2/3 (#9355), anti-phospho-T308-AKT (#9275), anti-Bcl-2 (#2870), anti-phospho-S102-YB1 (#2900) and anti-phospho-T202/Y204-MAPK (#4370) antibodies were obtained from Cell Signaling Technologies (Danvers, MA, USA). Anti-RSK2 (sc-9986), anti-p21 (sc-397), anti-RSK1 (sc-231), anti-MDM2 (SMP-14, sc-965) and anti-p53 (sc-126) antibodies were obtained from SantaCruz Biotechnology (Dallas, TX, USA). Mouse anti-α-tubulin (#T9026) was purchased from Sigma Aldrich (St. Louis, MO, USA). Horseradish peroxidase-conjugated secondary antibodies were obtained from Amersham Pharmacia Biotech (GE Healthcare Bio-Sciences Corporation, Piscataway, NJ, USA). The immunoblots were quantified with the ImageJ 1.53q program, and differences were evaluated against the amount of protein used to normalize. For each experiment, standard deviations were derived from three different measurements.

### 2.4. Cell Proliferation Assay

For cell proliferation assays, approximately the indicated number of cells were seeded in 60 mm dishes, and the day after, they were counted through direct observation with optical microscopy in triplicate to determine their actual number; wortmannin/BI-D1870/vehicle was then added. Triplicate plates were then counted at fixed times.

### 2.5. BrdU Assay

The bromodeoxyuridine (BrdU) kit was obtained from Boehringer Mannheim (Mannheim, Germany). In order to perform the analysis, a correct number of cells were placed on glass slides and incubated with bromodeoxyuridine, diluted in the culture medium (10 µmol/L), for 1 h. The slides were fixed and then permeabilized with an ethanol/glycine solution. They were then hybridized with a mouse monoclonal antibody against BrdU for 1 h and, after washing with 1× PBS, with a fluorescein (FITC)-conjugated secondary antibody for 1 h (Boehringer Mannheim, Mannheim, Germany). Finally, the hybridized slides were fixed on glass slides with 1× PBS-glycerol for microscope observation.

### 2.6. TUNEL Assay

To perform the TUNEL assay, approximately 100 × 10^3^ A549 or A375 cells were placed in a single well of a slide pretreated with L-polylysine (Corning Incorporated, Glendale, AZ, USA). They were then incubated for 72 h with vehicle or 4 µM BI-D1870. They were then fixed in solution of 4% paraformaldehyde and were subsequently permeabilized with a solution of 0.1% sodium citrate/0.1% Triton X-100. Slides were washed twice with PBS 1× for the TUNEL assay (Boehringer, Mannheim, Germany). All slides were also stained with 1 µg/mL Hoechst 33258 solution (Sigma Chemical Co., St. Louis, MO, USA), washed in PBS 1× and mounted with 1× PBS-glycerol. Fluorescence was detected using an epifluorescence microscope (Axiovert2, Carl Zeiss Vision GmbH, Munich, Germany) (10× objective) connected to KS300 analysis version 4.6 software (Zeiss). At least five different microscopic fields were analyzed, each containing at least 100 cells.

### 2.7. RNA Silencing

Small inhibitory RNA (siRNA) duplexes of the ON-target type plus SMARTpool were purchased from Dharmacon (Horizon Discovery Ltd., Cambridge, UK). Specifically, siRNAs were used to silence *RSK1* and *RSK2* (siRSK2 Human: #L-003026-00 and siRSK1 Human: #L-003025-00), and as a negative control, the non-targeting siCONTROL pool (#D-001206-13-05) was utilized. Transfections were performed using the DharmaFECT reagent with 100 nmol/L siRNA. After 24 h, cells were placed in 35 mm dishes at about 40% confluence in antibiotic-free culture medium with 10% FBS.

### 2.8. Immunohistochemistry and Histological Examination

All samples of melanomas, primary lung tumors and prostate tumors were collected from the “Ospedale del Mare” (ASL NA 1 Centro, Naples, Italy). Tumors were staged according to the TNM system of the American Joint Committee on Cancer (AJCC). By signing the informed consent form, the patients involved accepted to provide their tissue for scientific studies. The analyses were authorized by the Internal Reviewing Board. Tumor samples and corresponding normal tissue were fixed in a solution of 10% neutral buffered formalin and embedded in paraffin. The 4 μm sections were stained with hematoxylin/eosin according to standard procedures.

MDM2 expression was highlighted by staining the slides with the mouse anti-MDM2 antibody (clone IF2) #337100 (Invitrogen, Thermo Fisher Scientific, Waltham, MA, USA). RSK phosphorylation was checked by staining the slides with the mouse phospho-RSK2 antibody sc-374664 (SantaCruz Biotechnology, Dallas, TX, USA) for melanoma and the rabbit polyclonal phospho-RSK antibody sc-12445 (SantaCruz Biotechnology, Dallas, TX, USA) for prostate and lung tissues.

Immunoreactions were displayed using the ultraView Universal DAB Detection Kit (Ventana, Roche, Basel, Switzerland) with staining by the BenchMark IHC/ISH instrument for prostate and lung tissues and using the ultraView Universal Alkaline Phosphatase Red Detection Kit (Ventana, Roche, Basel, Switzerland) for melanoma with staining by BenchMark IHC/ISH instrument. Negative controls were performed by incubating samples in the absence of primary antibodies.

The sections were analyzed with an optical microscope (LEICA DM2000 LED, LEICA, Wetzlar, Germany). The images were acquired with a digital scanner (Ventana DP 200, Roche, Basel, Switzerland) at a magnification of 40×.

### 2.9. Statistical Analysis

Statistical analysis was performed using one-way ANOVA with standard parametric methods and Bonferroni’s multiple comparisons test (InStat program, GraphPad 3.1 software). Fisher’s exact test was used for χ2 analysis. SPSS 20 statistical software (SPSS Inc., IBM, New York, NY, USA) was used for data analysis. *p*-values less than 0.05 were considered statistically significant.

## 3. Results

### 3.1. Inhibition of p90RSK Is Able to Increase p53 Level via Reduction in MDM2 Phosphorylation at Serine 166

In a previously published manuscript [32], we demonstrated a novel mechanism by which p90RSK is capable of maintaining a low amount of p53, promoting its protein turnover through the phosphorylation of MDM2 at serine 166 and consequently promoting its proteasome-mediated degradation in follicular thyroid tumors [32]. Therefore, we decided to evaluate whether this mechanism was also confirmed in other types of tumors, in which the dysregulation of the MAPK pathway is directly involved in tumorigenesis.

**Figure 1:**
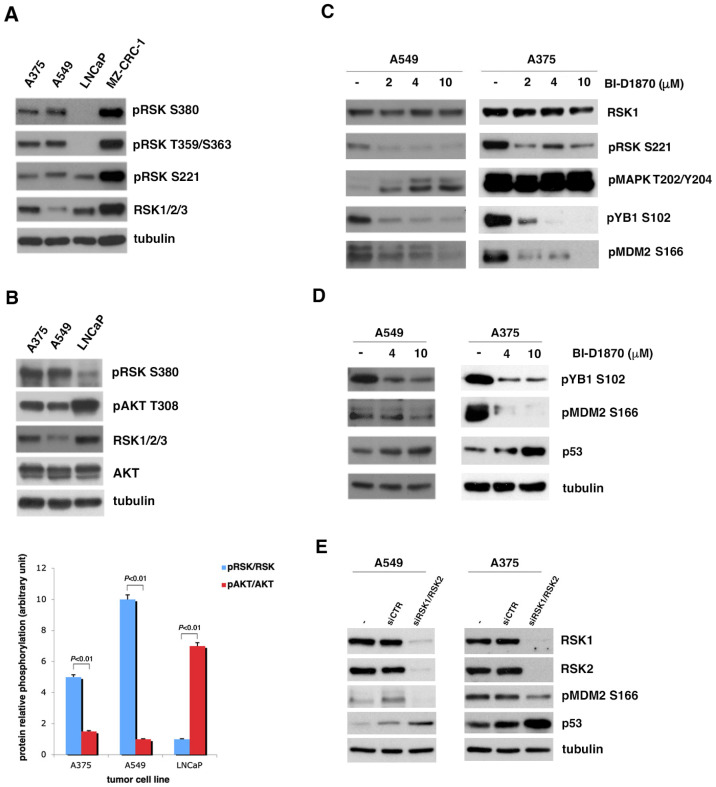
p90RSK induces p53 degradation by phosphorylating MDM2 at S166. (**A**) Several human tumor cell lines (A375, A549, LNCaP, MZ-CRC-1) were analyzed for the phosphorylation of p90RSK at different sites (pRSK S380, T359/S363, S221) and for the total level of p90RSK (RSK1/2/3). Tubulin was used to normalize. (**B**) The A375 the A549 cell lines, melanoma and lung cancer, respectively, were compared to the LNCaP prostate cancer cell line for the phosphorylation levels of p90RSK at S380 (pRSK S380) relative to total proteins (RSK1/2/3) and for the phosphorylation levels of AKT on T308 relative to total proteins (AKT). Tubulin was used for normalization (upper panel). The bar diagram below shows the ratio between the phosphorylated form and the total protein (pRSK/RSK and pAKT/AKT) obtained by quantifying each sample with the ImageJ program. Standard deviations were derived from three different quantifications (bottom panel). (**C**) A549 and A375 were incubated with the indicated doses of BI-D1870 for 1 h. The BI-D1870 inhibition efficacy was confirmed by the anti-phospho-S102 YB1 and anti-phospho-S221 RSK antibodies. Phosphorylated ERK1/2 antibody (pMAPK T202/Y204) was used to check the specificity of the inhibition. Phosphorylated MDM2 was visualized with anti-phospho-S166 MDM2 (pMDM2 S166) antibody. Anti-RSK1 antibody (RSK1) was used to normalize the immunoblot. (**D**) A549 and A375 cells were incubated with the indicated concentration of BI-D1870 inhibitor. The BI-D1870 effect was monitored by using the anti-phospho-S102 YB1 (pYB1 S102) antibody. Phosphorylated MDM2 was highlighted with anti-phospho-S166 MDM2 (pMDM2 S166) antibody. The p53 protein amount was determined using anti-p53 (p53) antibody. Anti-tubulin antibody was used to normalize the immunoblot. (**E**) A549 and A375 cells were transfected with a pool of specific RSK1/RSK2 siRNAs for interference against RSK1 and RSK2 or with control siCTR. To evaluate the efficiency of interference, we used anti-RSK1 and anti-RSK2 antibodies. The level of MDM2 phosphorylation was visualized with anti-phospho-S166 MDM2 antibody. The p53 level was determined by the use of anti-p53 antibody. Anti-MDM antibody was used for normalization. All experiments presented were repeated three times with consistent results. Furthermore, to also measure the relative contribution of AKT to MDM2 phosphorylation at S166, as described in literature [33], we analyzed the T308 phosphorylation of AKT in the A549 and A375 cells compared to that in the LNCaP cells (Figure 1B). It is immediately evident that in cells in which p90RSK is highly activated (A549 and A375 cell lines), AKT appears less phosphorylated than in the LNCaP cell line, which presents very active AKT, as expected due to PTEN mutation, but does not present strong phosphorylation of p90RSK.

To this end, using Western blotting, we analyzed the phosphorylation of p90RSK in different tumor cell lines with p53 in wild-type form to evaluate in which of them p90RSK was particularly active. As can be seen in Figure 1A, the strong phosphorylation of p90RSK at all phosphorylation sites significant for its activation [14,18,19] characterizes the A549 lung cancer cell line, the A375 melanoma cell line and the MZ-CRC-1 medullary thyroid carcinoma cell line. In contrast, p90RSK does not appear strongly phosphorylated in the LNCaP prostate cancer cell line, except for the low level of phosphorylation at S221.

Once we identified the cell lines in which p90RSK was very active, we decided to use BI-D1870, a pharmacological inhibitor of p90RSK, at different concentrations (2, 4 and 10 µM), to determine by Western blot which concentration was capable of inhibiting the phosphorylation of MDM2 at S166. In Figure 1C, it is evident that 4 µM of BI-D1870 is capable of preventing the activity of p90RSK, with a reduction in the phosphorylation at S221 of p90RSK causing not only a decrease in the phosphorylation of its canonical substrate YB1 at S106 [34] but also a decrease in the phosphorylation of MDM2 at S166 in both analyzed cell lines. Furthermore, the inhibition of p90RSK with BI-D1870 is noted to increase MAPK phosphorylation (pMAPK T202/Y204), suggesting that p90RSK controls the MAPK pathway with negative feedback to prevent the hyperactivation of the MAPK pathway [35].

Therefore, to explore whether the phosphorylation of MDM2 at serine 166 induced by p90RSK was important for the stability of p53 in the different cancer cell lines positive for p90RSK activation, as previously demonstrated for follicular thyroid cancer [32], these cells were incubated with different BI-D1870 concentrations (4 and 10 μM) for 1 h. In these cells, the turnover of p53 was regulated by MDM2, as p53 was in the wild-type form. The effect of BI-D1870 on p90RSK was checked by controlling YB1 phosphorylation at serine 102 (Figure 1D). In Figure 1D, it is evident that in all cell lines, both used concentrations of BI-D1870 induced a reduction in the phosphorylation of MDM2 at serine 166 and, consequently, an increase in the p53 amount. In the Supplementary Figure (Figure S1A), it is shown that this mechanism was also confirmed in the MZ-CRC-1 medullary thyroid carcinoma cell line, which is also characterized by a strong activation of p90RSK.

To verify that the increase in p53 depends on the ability of BI-D1870 to reduce the phosphorylation of MDM2 at S166 due to p90RSK inhibition independently of a possible non-specific stress effect of treatment, we performed gene silencing of p90RSK. So, we transfected A549 and A375 cells with a combination of different specific *RSK1/RSK2* siRNAs or with non-targeting siRNA (siCTR). A reduction in MDM2 phosphorylation at S166 and a corresponding increase in p53 levels were visible 48 h after transfection, supporting the hypothesis that p90RSK in these cells is capable of maintaining low p53 levels (Figure 1E).

### 3.2. In Tumor Cell Lines with Strongly Active p90RSK, MDM2-Mediated p53 Degradation Is Controlled by p90RSK

Based on the previous observations (Figure 1), we decided to study the effect of p90RSK-induced MDM2 phosphorylation at serine 166 on the stability of p53 at different times. We decided to use A375 and A549 as models of cell lines positive for p90RSK activation and LNCaP as negative control.

**Figure 2:**
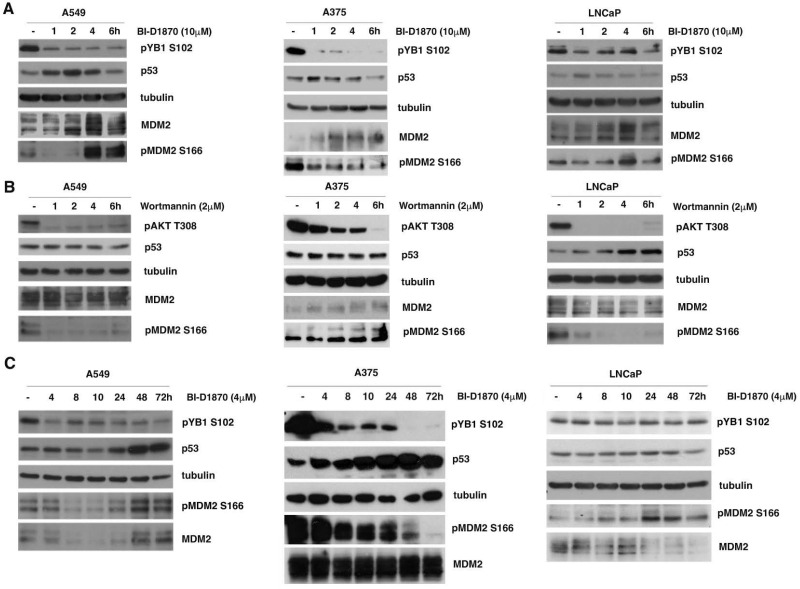
In tumor cell lines with strongly active p90RSK, MDM2-mediated p53 degradation is controlled by p90RSK. (**A**) A549, A375 and LNCaP cells were incubated with 10 μM BI-D1870 for the indicated times. The efficiency of the inhibition was confirmed by anti-phospho-S102 YB1 antibody. The immunoblot was checked by anti-p53, anti-MDM2 and anti-phospho-S166 antibodies, and anti-tubulin antibody was used for normalization. (**B**) A549, A375 and LNCaP cells were seeded in culture medium with 2 μM wortmannin for the indicated times. The efficiency of wortmannin was confirmed by using anti-phospho-T308 AKT antibody. The immunoblot was checked using anti-p53, anti-MDM2 and anti-phospho-S166 MDM2 antibodies, and normalization was carried out by immunoblotting with anti-tubulin antibody. (**C**) A549, A375 and LNCaP cells were seeded in culture medium with BI-D1870 to a 4 µM final concentration for the indicated times (h). The efficiency of the BI-D1870 was confirmed with anti-phospho-S102 YB1 antibody. The immunoblot was checked using anti-p53, anti-MDM2 and anti-phospho-S166 MDM2 antibodies and then normalized using tubulin. All presented experiments were repeated three times with consistent results.

A549, A375 and LNCaP cells were initially treated with 10μM BI-D1870 for short times (1, 2, 4, 6 h); in these cell lines, MDM2 controlled the turnover of p53, as it was expressed in wild-type form. The inhibition of p90RSK by BI-D1870 was checked by controlling the phosphorylation of YB1 at serine 102 (Figure 2A).

In A549 and A375 cells, following the use of BI-D1870 at a final concentration of 10μM for 1 and 2 h, a progressive reduction in S166-MDM2 phosphorylation was visible, which determines a rapid increase in p53 protein. Nevertheless, after 2, 4 and 6 h of incubation with BI-D1870, MDM2 also appeared to be overexpressed, most likely due to p53-dependent new MDM2 synthesized [32]. As a consequence of this increase, MDM2 seemed to be able to induce the degradation of p53 (Figure 2A, left and center panels). The results observed confirm the known regulatory feedback loop by which p53 transcriptionally activates its inhibitor MDM2, which is able to in turn promote the degradation of p53. The same results are clearly shown in the medullary thyroid carcinoma cell line MZ-CRC-1 (Figure S1B).

To formally demonstrate that BI-D1870 is also able to induce the regulatory feedback loop, we used Nutlin3a, an inhibitor of MDM2–p53 interaction. Nutlin3a was able to block the reduction in p53 levels, which appeared visible at 6 h in the absence of Nutlin3a (Figure S2).

On the contrary, in LNCaP cells, BI-D1870 was unable to strongly reduce the phosphorylation of MDM2 at S166 and consequently to greatly increase the level of p53, demonstrating that in this cell line, the MAPK/RSK pathway is not the main controller of MDM2-dependent p53 stabilization (Figure 2A, right panel).

To also establish the relative contribution of the PI3K/AKT pathway to the MDM2-mediated regulation of p53 in the considered cell lines, we treated the cells for different times (1, 2, 4, 6 h) with 2μM wortmannin, a potent inhibitor of phosphoinositide 3-kinase (PI3K). Surprisingly, the data obtained highlighted that in A549 and A375 cells, the suppression of AKT activity was not capable of significantly changing p53 levels, even when MDM2 was clearly dephosphorylated (Figure 2B, left and center panels). On the contrary, in LNCaP cells, a visible accumulation of p53 was already observable after four hours of treatment, confirming that in this cell line, the PI3K/AKT pathway is better able to control MDM2-dependent p53 degradation (Figure 2B, right panel).

Finally, we confirmed the increase in p53 protein, despite the consequent inhibition of p90RSK with BI-D1870 for long time intervals (4, 8, 10, 24, 48, 72 h) in A549 and A375 cell lines (Figure 2C, left and central panels). Prolonged MDM2 dephosphorylation at serine 166 caused by long durations of BI-D1870 treatment likely impaired the increase in MDM2 level by preventing it from degrading p53 (Figure 2C, left and center panels). The data were also confirmed in the medullary thyroid carcinoma cell line MZ-CRC-1 (Figure S1C).

From the data obtained, it can be concluded that p90RSK is efficient at down-regulating p53, allowing for its protein turnover in A549, A375 and MZ-CRC-1 cell lines, all characterized by strongly active p90RSK. In contrast, in LNCaP cells, which do not show strong p90RSK activation, this regulatory mechanism appears to be mainly controlled by the PI3K/AKT pathway.

### 3.3. p90RSK Is Efficient at Regulating Proliferation in A549 and A375 Cell Lines

**Figure 3:**
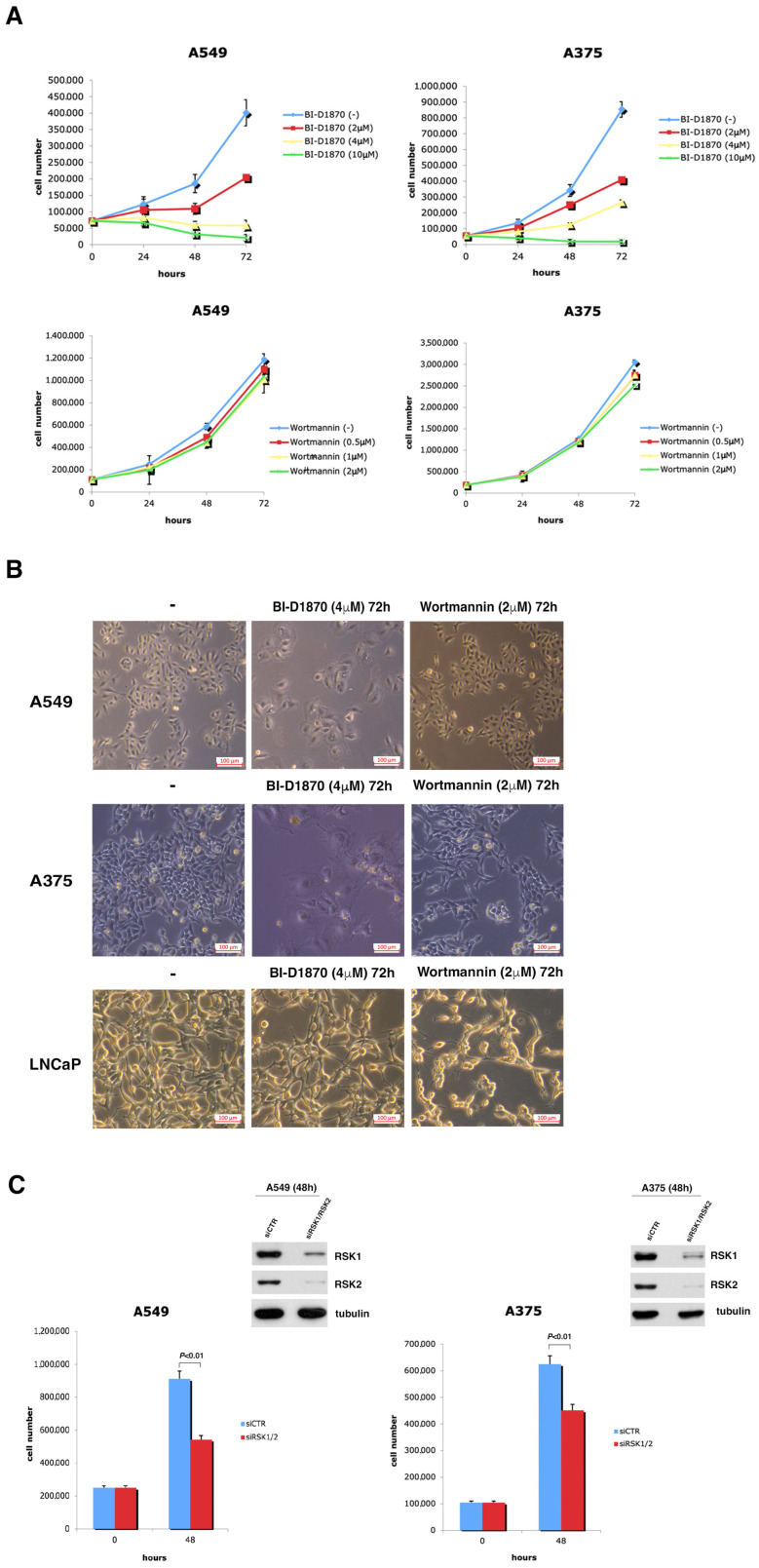
p90RSK is able to regulate rate of proliferation in A375 and A549 cell lines. (**A**) Growth curves of A375 and A549 cells seeded in culture medium with variable concentrations of BI-D1870. The experiments were repeated three times. The curves show the averages of three different measurements ± standard deviation. (**B**) A375, A549 and LNCaP cells, treated or not with 4 µM BI-D1870 or with 2 µM wortmannin for 72 h, were photographed in the optical field at 10× magnification. A representative area is shown. (**C**) Growth curves of A549 and A375 cells transfected with a combination of different RSK1/RSK2 specific siRNA or with non-targeting siRNA (siCTR). The efficiency of transfection was measured with anti-RSK1 and anti-RSK2 antibodies. Normalization was performed with an antibody against tubulin. The experiments were conducted three different times. The histograms show the average value of the obtained results at 0 and 48 h and the standard deviation.

First of all, we tested the effect of p90RSK activity on cell growth by plotting a proliferation curve of A549 and A375 cells incubated with culture medium supplemented with BI-D1870 (2, 4, 10 µM). The obtained data make evident a considerable diminution in cell number due to the inhibition of p90RSK; in fact, following 72 h of incubation with 2 µM of BI-D1870, the number of A375 cells was less than half (408,982) compared to that in the untreated control (853,672), and the number of A549 cells was just over half (203,917) compared to that in the untreated control (400,833), exhibiting an anti-proliferative effect of BI-D1870 at a low dose on both cell lines (Figure 3A, upper panels).

The inhibition of cell growth by BI-D1870 was also confirmed in the medullary thyroid carcinoma cell line MZ-CRC-1 (Figure S1D).

In contrast, the growth curves in the lower panels of Figure 3A demonstrate that wortmannin had a minor effect on cell proliferation, consistent with the fact that in A375 and A549 cells, the MDM2-dependent regulation of p53 is mainly controlled by the MAPK/p90RSK pathway and not by the PI3K/AKT pathway.

The effect of p90RSK inhibition due to BI-D1870 (4 µM/72 h) treatment on the growth of A375 and A549 cells is evident in Figure 3B, upper and center panels, where light microscopy images of representative fields are shown. In both cell lines, treatment with BI-D1870 caused a decrease in the number of cells, which also appeared less birefringent, while treatment with wortmannin showed only a very slight effect on the cell number. Therefore, these images confirm what was seen in the A375 and A549 cell growth curves. In contrast, in LNCaP cells, the inhibition of p90RSK and AKT by the specific inhibitors had approximately the opposite effect on cell growth compared to that obtained for the other cell lines analyzed (Figure 3B, bottom panel).

Moreover, to demonstrate that p90RSK was directly implicated in the control of cell growth, we performed silencing with a pool of siRNA (siRSK1/RSK2) in A375 and A549 cell lines (Figure 3C). The data obtained show that the genetic inhibition of p90RSK after 48 h was able to significantly reduce the number of cells in both cell lines.

### 3.4. In A459 and A375 Cells, p90RSK Controls DNA Synthesis and Apoptosis through Regulation of p53

Since we have shown that in A549 and A375 cells, p53 increases due to the progressive dephosphorylation of MDM2 at S166, and knowing that p53 is a regulator of the transcription of various genes such as the promotor of cell cycle arrest *p21* [36] and the antiapoptotic gene *Bcl-2* [37], we decided to determine by Western blot analysis whether a change in *p21* and *Bcl-2* expression occurred in these cells due to treatment with BI-D1870 for 24, 48 and 72 h.

**Figure 4:**
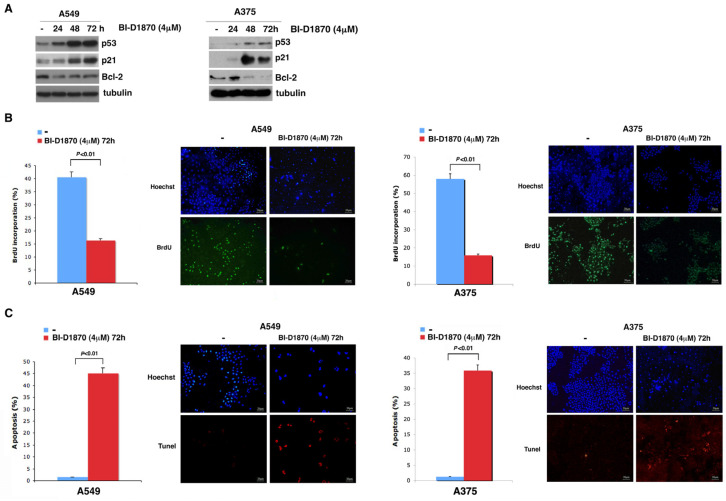
p90RSK is capable of controlling DNA synthesis and apoptosis by regulating p53 levels. (**A**) A549 and A375 cells were incubated with 4 μM BI-D1870 for the indicated times (h). The immunoblot was checked using anti-p21, anti-Bcl2, anti-Bax and anti-p53 antibodies; the anti-tubulin antibody was used for normalization. (**B**) BrdU assay in A549 and A375 cells incubated or not with 4 μM BI-D1870 for 72 h. We used Hoechst solution to stain the nuclei blue, and anti-BrdU antibody was used to highlight apoptotic cells (green stain). In each sample, we counted at least 500 cells. The percentage of BrdU-positive cells was obtained from the ratio of BrdU-labeled nuclei to total nuclei. The histogram shows the average of the counts performed in different fields of BrdU-stained cells (more than 100 cells were counted in each field). The experiment was conducted in triplicate. The standard deviation of the acquired measurements was determined (left panel). The images in the figure represent photographs obtained with a fluorescence microscope at 10× magnification. A representative area for each sample is shown in the figure (right panel). (**C**) TUNEL assay in A549 and A375 cells treated or not with 4 μM BI-D1870 for 72 h. Hoechst solution was used to stain the nuclei blue, and apoptotic cells were stained using the TUNEL assay (red staining). The percentage of apoptotic cells was evaluated from the ratio of red-stained nuclei to blue-stained nuclei. The histogram shows the average of number of apoptotic cells counted in several fields (more than 500 cells were counted in total) and the standard deviation of the values obtained (left panel) in three different experiments. The images in the figure represent photographs obtained with a fluorescence microscope at 10× magnification. One representative field for is sample is displayed (right panel).

In Figure 4A, it is evident that in both A549 and A375 cell lines, treatment with 4μM BI-D1870 for 24, 48 and 72 h induced an accumulation of p21 and a decrease in Bcl-2, both plausibly dependent on the increase in p53.

To study the effect exerted by the p53-dependent p21 increase on cell replication, we performed a BrdU assay, in which A549 and A375 cells were treated with BrdU for 1 h after 72 h of treatment with 4 µM BI-D1870. It is clearly evident that A375 cells treated with BI-D1870 incorporated a smaller amount of BrdU (15.83%) than untreated cells (58%), and treated A549 cells incorporated less BrdU (16.3%) than untreated cells (40.6%) (Figure 4B). This is consistent with a reduction in cell replication dependent on the increase in the cell cycle inhibitor p21 due to p90RSK inhibition.

Furthermore, to demonstrate that the p53-dependent reduction in Bcl-2 following p90RSK inhibition actually induced an increase in apoptosis in the A375 and A549 cell lines, we performed a TUNEL assay, in which we treated both cell lines for 72 h with 4 μM BI-D1870. This treatment induced an increase in the apoptosis rate of A375 cells (35.92%) compared to that of untreated cells (1.24%) and also of A549 cells (45.12%) compared to that of the untreated control (1.49%) (Figure 4C).

### 3.5. p90RSK Phosphorylation Is Correlated with Stabilization of MDM2 in Primary Lung Tumors and Melanomas

Previously published data demonstrated a clear increase in the stabilization of MDM2 in thyroid follicular primary tumors in which the p90RSK kinase was activated by increased phosphorylation [32]. So, we analyzed the potential association between activated p90RSK (pRSK) and MDM2 accumulation (MDM2) in primary lung cancers and melanomas.

**Figure 5:**
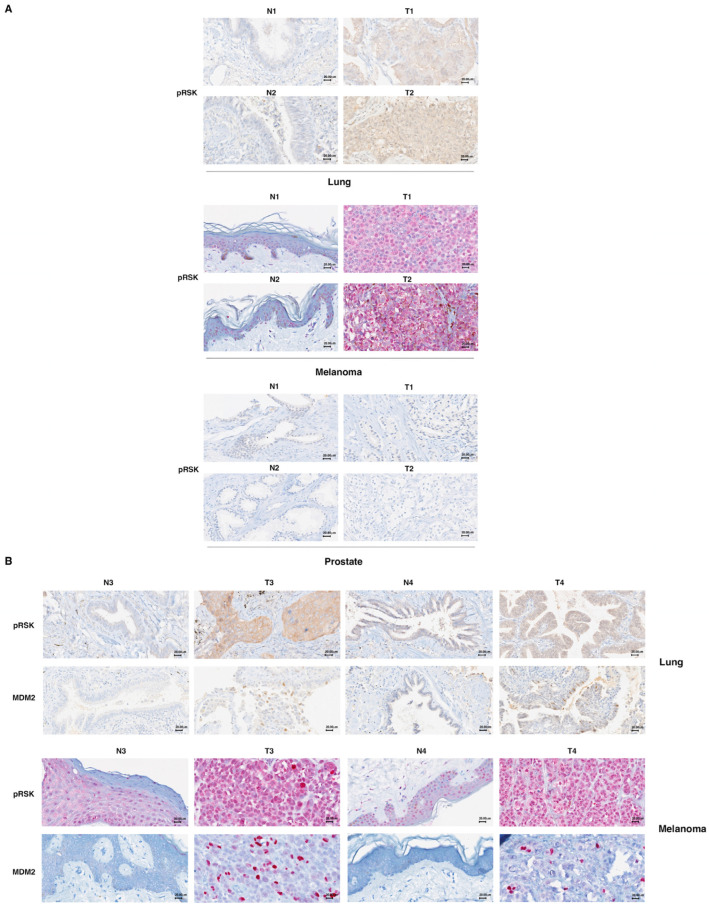
MDM2 stabilization is associated with p90RSK activation in primary melanomas and primary lung tumors. (**A**) Formalin-fixed, paraffin-embedded normal (N) and tumor (T) tissue samples were stained using an immunohistochemical technique for RSK phosphorylation. To this end, normal (N) or tumor (T) tissue samples were incubated with anti-pRSK antibodies. A diffuse cytoplasmic/nuclear positivity for pRSK characterized the T samples; in contrast, N samples present a reduction in the percentage of positive cells or only a weak positivity. For each tumor type (lung, melanoma and prostate), we show two representative images of different samples related to the samples (both T and N) shown in Supplementary Tables S1 and S2. 40× magnification. (**B**) Formalin-fixed, paraffin-embedded normal (N) and tumor (T) tissue samples were stained using an immunohistochemical technique for MDM2 and pRSK. Each tissue sample (both T and N) was stained with anti-MDM2 and anti-pRSK antibodies. Diffuse cytoplasmic/nuclear evident detection for pRSK is evident in tumor samples (T), while samples from normal lung and skin tissues (N) were less positive. Diffuse nuclear positivity to MDM2 staining, indicating its nuclear accumulation, characterizes the T samples, while the corresponding N samples are negative. Representative images of the samples indicated in Supplementary Tables S1 and S2 are shown. 40× magnification.

To this end, we performed immunohistostaining for pRSK and MDM2 in 12 primary lung tumors, 12 melanomas and five prostate tumors, comparing each to its normal counterpart (Figure 5A).

First, we tested whether primary lung cancer and melanoma, compared to their normal tissues, exhibited an increase in the phosphorylation of p90RSK (pRSK); the same test was performed for prostate cancer. In the 12 primary lung tumors analyzed, 8 were positive for increased phosphorylation of p90RSK (66.7%); in the 12 melanomas analyzed, 10 were positive for increased phosphorylation of p90RSK in tumor tissues compared to normal counterparts (83.3%); all five prostate tumors were negative for increased phosphorylation of p90RSK (100%).

Thereafter, we analyzed the correspondence between the positivity for increased p90RSK phosphorylation and MDM2 accumulation in analyzed tumors (Figure 5B, Supplementary Tables S1 and S2). In the analyzed primary lung tumors, we found eight tumors positive for both pRSK and MDM2 (100% match); four tumors were negative for both pRSK and MDM2 staining (100% match) (Supplementary Table S1). In the analyzed melanomas, we found seven tumors positive for both pRSK and MDM2 (100% match); two tumors were negative for both pRSK and MDM2 staining (100% match). Only three melanomas were positive for an increase in pRSK but negative for MDM2 (25% of total) (Supplementary Table S2). This possibility could be explained by the existence of p53 mutations found in 30% of cases of cutaneous melanomas [38]. Characteristic of the p53 mutants is their high expression due to greater stability; mutant p53 is in fact incapable of inducing the expression of its primary regulator, MDM2 [39].

The analysis with Fisher’s exact statistical test demonstrated that the analyzed primary tumors showed an appreciable correlation between phosphorylated p90RSK and MDM2 expression. The percentage of MDM2-positive tumors was significantly higher in the group with stronger p90RSK phosphorylation; however, the percentage of negativity for MDM2 was considerably higher in tumors negative for an increase in p90RSK phosphorylation (*p* < 0.05).

Tumors were considered positive for increased p90RSK phosphorylation when they had an increase in staining of at least 10% of total cells and a clear increase in staining intensity compared to the normal counterparts. Tumors were defined as positive for MDM2 overexpression in the presence of nuclear staining in ≥5% of total cells compared to normal tissues that were always negative for staining (0% of total cells).

## 4. Discussion

Many human tumors are characterized by activating mutations in kinases belonging to the MAPK pathway, which in turn make the MAP pathway constitutively activated [40,41,42,43,44,45,46,47,48,49]. For this reason, in the last decade, various pharmacological inhibitors of the MAPK pathway have been used, directed against mutated BRAF (first generation RAF inhibitors as vemurafenib, encorafenib, dabrafenib, sorafenib) or MEK (binimetinib, cobimetinib, trametinib) in tumor therapy [50,51,52,53]. They show good tolerability, with relatively mild toxicity and manageable side effects. Nevertheless, one of the disadvantages of BRAF inhibitors is that they are specifically effective in tumors with mutated BRAF such as melanomas, since in those with BRAFwt, a “paradoxical activation” of ERK signaling may occur due to the drug-mediated transactivation of RAF dimers that in turn can induce other types of cancers, mainly skin cancers. In contrast, in tumors with mutated BRAF, RAS is not activated, so transactivation due to the drug is very low [54]. The second important issue is that although in the first period of treatment, the use of these inhibitors yields a good therapeutic response, mechanisms of resistance usually develop within six to eight months of treatment in approximately 50% of patients [55,56]. In most cases, these resistance mechanisms lead to a restart of the MAPK pathway [57,58,59,60]. Accordingly, the search for new effective molecular targets is a priority for the improvement of tumor therapies [61,62].

In particular, molecules further downstream in the MAPK pathway could be valid new targets. In fact, by targeting these molecules, the anti-tumor efficacy should be improved, as the development of resistance mechanisms associated with their inhibition should be significantly reduced. For example, the use of ERK1/2 inhibitors in melanoma cells has shown good efficacy in inhibiting the MAPK pathway and cell proliferation, although with some limitations; clinical studies have further validated their usefulness (e.g., NCT01781429, NCT01875705) [63,64,65]. Even in non-small cell lung cancers, a role for ERK inhibitors in combination with the anti-tumor activity of natural cytotoxic molecules has been demonstrated [66].

It has been demonstrated that low doses with better tolerability do not always yield a good therapeutic response. However, these drugs have shown a high toxicity profile when used at doses with good efficacy, probably justified by the involvement of the MAPK transduction pathway in many essential cellular processes. The side effects observed are comparable to those of other MAPK pathway inhibitors, such as increased liver function tests, diarrhea, nausea, vomiting, hyponatremia, pruritus, increased amylase, anemia and rash [67,68,69].

Moreover, some ERK1/2 inhibitors experience resistance after the first period of administration [70], probably due to their mechanism of action. These inhibitors block ERK1/2 in the cytoplasm and not in the nucleus [71]. Thus, their negative feedback loop on MAPKs, necessary to balance pathway activity by reducing the expression of upstream kinases, is interrupted. This leads to the hyperactivation of upstream elements, which establishes a drug resistance mechanism [70].

So, researchers are currently focused on identifying specific inhibitors that target effectors further downstream in the MAPK pathway with a more selective role, necessary to minimize the adverse effects associated with their inhibition. In this regard, members of the p90RSK family are particularly interesting. They have a very low mutation rate in tumors (≤2%) and phosphorylate several substrates involved in tumorigenesis [35]. An ever-increasing number of tumors showing the hyperactivation of p90RSK has been identified, and the development of inhibitors that target p90RSK may become a valuable and more specific alternative to the use of already available MAPK pathway inhibitors [72,73].

Despite the fact that p90RSK is recognized as playing an important role in the progression of cancer, the development of specific p90RSK inhibitors is slower compared to the development of inhibitors targeting other members of the MAPK signaling pathway. In fact, at this stage, none of these compounds have entered advanced-stage clinical trials [35].

In this manuscript, we have demonstrated that in tumors characterized by a very active p90RSK, p90RSK inhibition induces a replication block and promotes apoptosis due to RSK/MDM2-dependent p53 stabilization. Although only a limited number of tumor samples were analyzed so far, the data obtained highlight an important role of p90RSK inhibition in blocking the progression of tumorigenesis, supporting researchers’ extensive efforts in the development of specific p90RSK inhibitors.

p53 is a transcription factor that controls the transcription of many target genes, playing a very relevant role in the regulation of the cell cycle, apoptosis and genome stability [74,75]; for this reason, it is defined as the guardian of the genome [76]. In many pathologies and especially in tumors, *p53* presents numerous inactivating genetic mutations that favor tumorigenesis and that make it a very interesting target for the development of new anti-tumor therapies [77,78]. Here, we have demonstrated that targeting the p90RSK/MDM2/p53 pathway is effective in blocking tumors with oncogenic up-regulation of the MAPK pathway such as melanoma and lung cancer. In fact, the overactivation of p90RSK is capable of maintaining very low levels of wild-type p53 through the phosphorylation of MDM2 at S166 and its stabilization.

**Figure 6:**
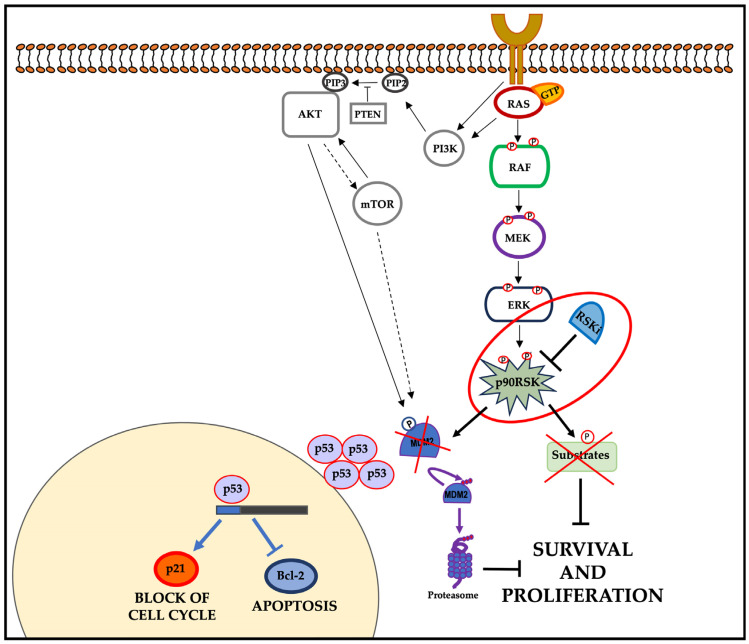
Final model: the inhibition of p90RSK induces the suppression of proliferation and survival. In tumor cells characterized by constitutive oncogenic activation of the ERK pathway, p90RSK inhibition destabilizes MDM2, inducing its proteasome degradation. The MDM2 reduction leads to an increase in p53 that, in turn, induces p21 transcription and reduces Bcl-2 transcription. The PI3K/AKT pathway is able to contribute to this mechanism in tumor cells where it is overactive.

Therefore, p90RSK inhibitors could act in two different ways simultaneously: by blocking the downstream MAPK pathway and by acting indirectly on wild-type p53 function. In fact, by destabilizing the p53/MDM2 complex due to the RSK-dependent phosphorylation of MDM2, the degradation of p53 is prevented, with a consequent increase in its protein level; in this way, the protein can act as a transcriptional factor, activating apoptosis and blocking the cell cycle (Figure 6).

Furthermore, the combination of p90RSK inhibitors with other agents that act on targets of the same or other pathways could improve the therapeutic response.

An involvement of p90RSK in metabolism has been demonstrated [79]. Furthermore, a close correlation has been shown between oncogenic KRAS expression and pleiotropic metabolic changes that occur as primary events supporting tumorigenesis and subsequently sustaining tumor progression. Such metabolic adaptations make tumor cells dependent on nutrient supply, offering new therapeutic opportunities [80].

Since the aim of this study, by identifying a new possible molecular target, is to provide the possibility of administering new combined therapies in tumors with an activated MAPK pathway, it could be interesting to include among future objectives the study of a possible synergistic effect between the drugs targeting tumor metabolism and inhibitors of the MAPK pathway targeting p90RSK.

## 5. Conclusions

In this manuscript, we have demonstrated that in tumors characterized by strongly active p90RSK, p53 has low cellular levels due to the p90RSK-dependent phosphorylation of MDM2 at S166 and subsequent stabilization of MDM2. Furthermore, p53 controls the proliferation and apoptosis of the A375 malignant melanoma cell line and the A549 lung tumor cell line, both characterized by the strong activation of p90RSK, by transcriptionally controlling of the expression of the cell cycle inhibitor *p21* and the antiapoptotic gene *Bcl-2*. Finally, we have highlighted the existence of a significant correlation between p90RSK activation and MDM2 stabilization in primary lung tumors and melanomas.

## Data Availability

Data are contained within the article and Supplementary Materials.

## Supplementary Materials

The following supporting information can be downloaded at: https://www.mdpi.com/article/10.3390/cells13181546/s1, Figure S1: The p90RSK-mediated control of p53 levels via the phosphorylation of MDM2 at S166 contributes to the regulation of cell growth in MZ-CRC-1 cells, Figure S2: Nutlin3a is able to block the MDM2-dependent reduction in p53 levels during treatment with BI-D1870. Table S1: The results of immunohistochemical evaluations for each normal (N) and tumor (T) lung specimens (1 to 12), Table S2: The results of immunohistochemical evaluations for each normal (N) and tumor (T) specimen for melanoma sections (1 to 12).
