# Supplementary material for: Targeting the p90RSK/MDM2/p53 Pathway Is Effective in Blocking Tumors with Oncogenic Up-Regulation of the MAPK Pathway Such as Melanoma and Lung Cancer
Source: Cells. 2024 Sep 14;13(18):1546. doi: 10.3390/cells13181546 (PMC11430938; doi:10.3390/cells13181546)
Supplement: Supplementary file 1 [file cells-13-01546-s001.zip › cells-3175937-supplementary.pdf]

cells

MDPI

Supplementary Materials

Targeting the p90RSK/MDM2/p53 Pathway Is Effective in Blocking Tumors with Oncogenic Up-Regulation of the MAPK Pathway Such as Melanoma and Lung Cancer

Immacolata Maietta [1], Eleonora Viscusi [2], Stefano Laudati [3], Giuseppe Iannaci [2], Antonio D'Antonio [3], Rosa Marina Melillo [1,4], Maria Letizia Motti [5,*,†] and Valentina De Falco [1,*,†]

[1] Institute of Endocrinology and Experimental Oncology (IEOS), National Research Council (CNR), Via S. Pansini 5, 80131 Naples, Italy; immacolata.maietta@uvigo.gal (I.M.); rosmelil@unina.it (R.M.M.)
[2] U.O.C. Anatomia Patologica, P.O. Pellegrini ASL NA1 Centro, 80134 Naples, Italy; eleonora.viscusi@libero.it (E.V.); giuseppe.iannaci@aslnapoli1centro.it (G.I.)
[3] U.O.C. Anatomia Patologica, Ospedale del Mare ASL NA1 Centro, 80147 Naples, Italy; stefano.laudati@aslnapoli1centro.it (S.L.); antonio.dantonio@aslnapoli1centro.it (A.D.)
[4] Department of Molecular Medicine and Medical Biotechnology, University of Naples Federico II, 80131 Naples, Italy
[5] Department of Medical, Movement and Wellbeing Sciences, University of Naples Parthenope, 80133 Naples, Italy
* Correspondence: letizia.motti@uniparthenope.it (M.L.M.); valentina.defalco@cnr.it (V.D.F.)
† These authors contributed equally to this work.

Supplementary Materials: The following supporting information can be downloaded at: www.mdpi.com/xxx/s1, Figure S1: p90RSK-mediated control of p53 levels via phosphorylation of S166 MDM2 contributes to the regulation of cell growth in MZ-CRC-1 cells, Figure S2: Nutlin3a is able to block the MDM2-dependent reduction in p53 levels during treatment with BI-D1870. Table 1: The results of immunohistochemical evaluations for each normal (N) and tumor (T) lung specimens (1 to 12), Table 2: The results of immunohistochemical evaluations for each normal (N) and tumor (T) specimen for melanoma sections (1 to 12).

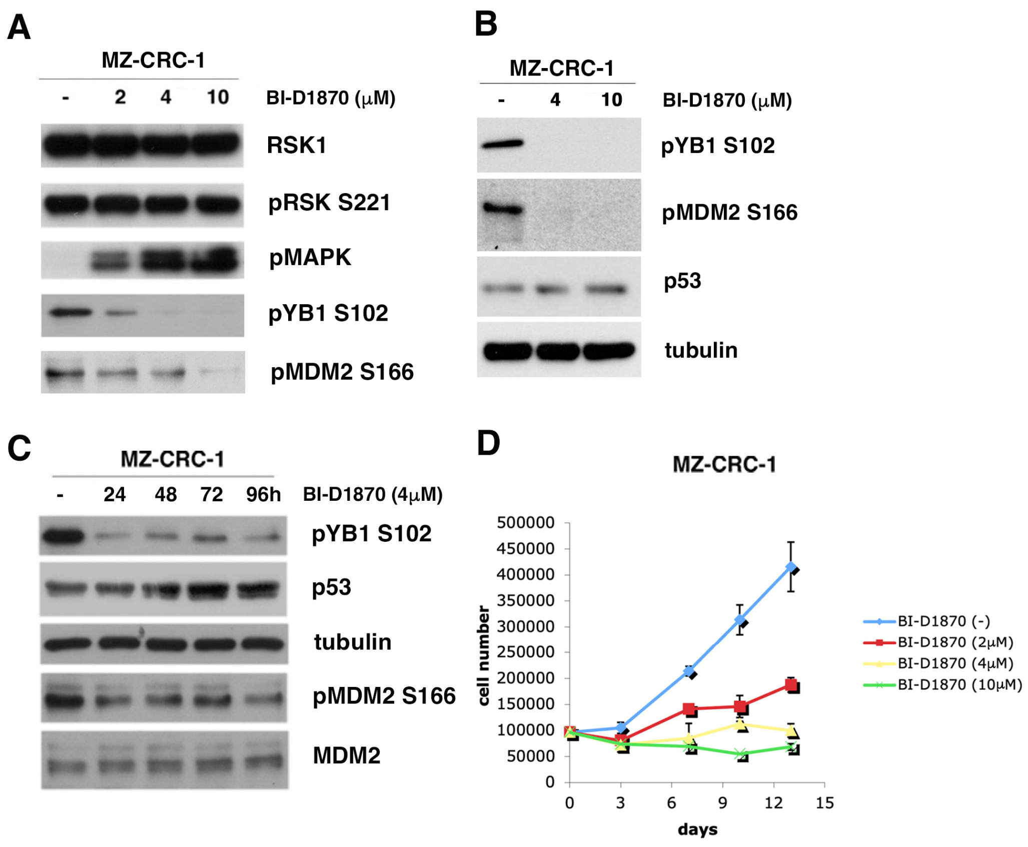

Figure S1. p90RSK-mediated control of p53 levels via phosphorylation of S166 MDM2 contributes to the regulation of cell growth in MZ-CRC-1 cells. (**A**) MZ-CRC-1 cells were treated with the indicated doses of BI-D1870 for 1h. The BI-D1870 inhibition efficiency was tested with the anti-phospho-S102 YB1 and with the anti-phospho-S221 RSK antibodies. Phosphorylated MAPK (pMAPK) was used to check the specificity of the inhibition. Phosphorylated MDM2 was revealed with anti-phospho-S166 MDM2 (pMDM2 S166) antibody. Normalization was performed by immunoblotting with anti-RSK1 (RSK1) antibody. (**B**) MZ-CRC-1 cells were treated with the indicated doses of BI-D1870 for 1h. BI-D1870 inhibition efficiency was tested with the anti-phospho-S102 YB1 (pYB1 S102) antibody. Phosphorylated MDM2 was revealed with anti-phospho-S166 MDM2 (pMDM2 S166) antibody. p53 total level was revealed with anti-p53 (p53) antibody. Normalization was performed by immunoblotting with anti-tubulin antibody. (**C**) MZ-CRC-1 cells were treated with 4 μM BI-D1870 for the indicated times (h). The efficacy of the treatment was verified with anti-phospho-S102 YB1 antibody. The immunoblot was then tested with anti-p53, anti-MDM2, anti-phospho-S166 MDM2 antibodies and normalized for tubulin. (**D**) Growth curves of MZ-CRC-1 cells treated with different doses of BI-D1870. The experiment was conducted in triplicate. The results in the histogram are the average of the three independent determinations +/- standard deviation.

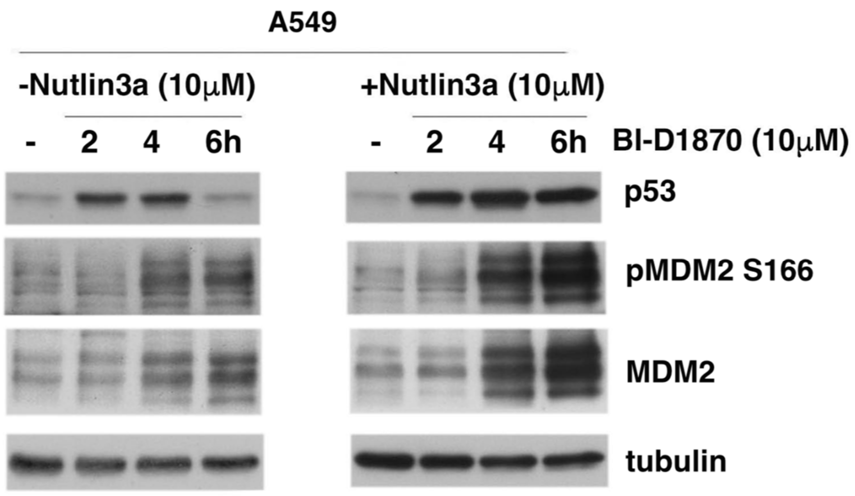

Figure S2. Nutlin3a is able to block the MDM2-dependent reduction in p53 levels during treatment with BI-D1870. A549 cells were treated with BI-D1870 10μM with or without Nutlin3a at the indicated times. The immunoblot was tested with anti-p53, anti-MDM2 and anti-phospho-S166 MDM2 antibodies and normalized with anti-tubulin.

Sample	pRSK Antibody	Total MDM2 Antibody	Sample	pRSK Antibody	Total MDM2 Antibody
1	**N**: 0%<br>NO SIGNAL<br><br>**T**: 100%<br>CYTOSOLIC<br>MODERATE	**N**: 0%<br>NO SIGNAL<br><br>**T**: 5%<br>NUCLEAR<br>MODERATE	7	**N**: 0%<br>NO SIGNAL<br><br>**T**: 40%<br>CYTOSOLIC<br>WEAK	**N**: 0%<br>NO SIGNAL<br><br>**T**: 5%<br>NUCLEAR<br>MODERATE
2	**N**: 0%<br>NO SIGNAL<br><br>**T**: 100%<br>CYTOSOLIC<br>MODERATE	**N**: 0%<br>NO SIGNAL<br><br>**T**: 5%<br>NUCLEAR<br>MODERATE	8	**N**: 0%<br>NO SIGNAL<br><br>**T**: 100%<br>CYTOSOLIC<br>WEAK/MODERATE	**N**: 0%<br>NO SIGNAL<br><br>**T**: 5%<br>NUCLEAR<br>MODERATE
3	**N**: 0%<br>NO SIGNAL<br><br>**T**: 100%<br>CYTOSOLIC<br>STRONG	**N**: 0%<br>NO SIGNAL<br><br>**T**: 5%<br>NUCLEAR<br>MODERATE/STRON	9	**N**: 0%<br>NO SIGNAL<br><br>**T**: 0%<br>NO SIGNAL	**N**: 0%<br>NO SIGNAL<br><br>**T**: 0%<br>NO SIGNAL
4	**N**: 20%<br>CYTOSOL<br>WEAK<br><br>**T**: 100%<br>CYTOSOLIC<br>MODERATE/STRONG	**N**: 0%<br>NO SIGNAL<br><br>**T**: 8%<br>NUCLEAR<br>MODERATE/STRONG	10	**N**: 0%<br>NO SIGNAL<br><br>**T**: 0%<br>NO SIGNAL	**N**: 0%<br>NO SIGNAL<br><br>**T**: 0%<br>NO SIGNAL
5	**N**: 0%<br>NO SIGNAL<br><br>**T**: 100%<br>CYTOSOLIC<br>WEAK	**N**: 0%<br>NO SIGNAL<br><br>**T**: 25%<br>NUCLEAR<br>STRONG	11	**N**: 0%<br>NO SIGNAL<br><br>**T**: 0%<br>NO SIGNAL	**N**: 0%<br>NO SIGNAL<br><br>**T**: 2%<br>NUCLEUS<br>MODERATE
6	**N**: 0%<br>NO SIGNAL<br><br>**T**: 30%<br>CYTOSOLIC<br>WEAK	**N**: 0%<br>NO SIGNAL<br><br>**T**: 5%<br>NUCLEAR<br>WEAK/MODERATE	12	**N**: 0%<br>NO SIGNAL<br><br>**T**: 0%<br>NO SIGNAL	**N**: 0%<br>NO SIGNAL<br><br>**T**: 0%<br>NO SIGNAL

Table S1. The results of immunohistochemical evaluations for each normal (N) and tumor (T) specimen for lung samples (1 to 12) have been shown in the table. Results are displayed based on the following parameters:

- % of colored cells out of the total cells counted
- signal evaluation (cytosolic, nuclear)

- color intensity (weak, moderate, strong)

Tumor samples have been considered positive if the percentage of staining compared to the normal counterpart was >10% (due to RSK phosphorylation) or >=5% (due to MDM2 nuclear expression) and if the staining intensity was stronger in the tumor compared to the corresponding normal counterpart.

Samples 1,2,3,4,5,6,7,8 are positive for increased RSK phosphorylation and nuclear MDM2 overexpression.

Samples 9,10,11,12 are negative for increased RSK phosphorylation and nuclear MDM2 overexpression.

Sample	pRSK Antibody	Total MDM2 Antibody	Sample	pRSK Antibody	Total MDM2 Antibody
1	N: 50%/25%<br>CYTOSOLIC/NUCLEAR<br>WEAK/MODERATE<br><br>T: 80%/50%<br>CYTOSOLIC/NUCLEAR<br>MODERATE/STRONG	N: 0%<br>NO SIGNAL<br><br>T: 5%<br>NUCLEAR<br>MODERATE	7	N: 60%<br>CYTOSOLIC/NUCLEAR<br>MODERATE/STRONG<br><br>T: 95%<br>CYTOSOLIC/NUCLEAR<br>MODERATE/STRONG	N: 0%<br>NO SIGNAL<br><br>T: 10%<br>NUCLEAR<br>MODERATE
2	N: 40%<br>NUCLEAR<br>WEAK<br><br>T: 90%<br>CYTOSOLIC/NUCLEAR<br>MODERATE/STRONG	N: 0%<br>NO SIGNAL<br><br>T: 10%<br>NUCLEAR<br>MODERATE/STRONG	8	N: 80%<br>NUCLEAR<br>MODERATE/STRONG<br><br>T: 90%<br>CYTOSOLIC/NUCLEAR<br>MODERATE/STRONG	N: 0%<br>NO SIGNAL<br><br>T: 5%<br>NUCLEAR<br>MODERATE
3	N: 70%<br>CYTOSOLIC/NUCLEAR<br>WEAK<br><br>T: 80%<br>CYTOSOLIC/NUCLEAR<br>STRONG	N: 0%<br>NO SIGNAL<br><br>T: 30%<br>NUCLEAR<br>MODERATE/STRONG	9	N: 50%<br>CYTOSOLIC/NUCLEAR<br>WEAK/MODERATE<br><br>T: 80%<br>CYTOSOLIC/NUCLEAR<br>WEAK/STRONG	N: 0%<br>NO SIGNAL<br><br>T: <1%<br>NUCLEAR<br>MODERATE
4	N: 50%<br>CYTOSOLIC/NUCLEAR<br>WEAK<br><br>T: 100%<br>CYTOSOLIC/NUCLEAR<br>MODERATE/STRONG	N: 0%<br>NO SIGNAL<br><br>T: 20%<br>NUCLEAR<br>STRONG	10	N: 70%<br>NUCLEAR<br>WEAK/MODERATE<br><br>T: 90%<br>CYTOSOLIC/NUCLEAR<br>MODERATE/STRONG	N: 0%<br>NO SIGNAL<br><br>T: 10%<br>NUCLEAR<br>MODERATE
5	N: 90%<br>CYTOSOLIC/NUCLEAR<br>WEAK/STRONG<br><br>T: 90%<br>CYTOSOLIC/NUCLEAR<br>WEAK/STRONG	N: 0%<br>NO SIGNAL<br><br>T: < 1%<br>NUCLEAR<br>WEAK	11	N: 80%<br>CYTOSOLIC/NUCLEAR<br>WEAK/MODERATE<br><br>T: 90%<br>CYTOSOLIC/NUCLEAR<br>MODERATE/STRONG	N: 0%<br>NO SIGNAL<br><br>T: <1%<br>NUCLEAR<br>WEAK
6	N: 100%<br>CYTOSOLIC/NUCLEAR<br>WEAK/STRONG<br><br>T: 100%<br>CYTOSOLIC/NUCLEAR<br>WEAK/STRONG	N: 0%<br>NO SIGNAL<br><br>T: 1%<br>NUCLEAR<br>WEAK	12	N: 40%<br>NUCLEAR<br>WEAK/MOD<br><br>T: 90%<br>CYTOSOLIC/NUCLEAR<br>WEAK/STRONG	N: 0%<br>NO SIGNAL<br><br>T: <1%<br>NUCLEAR<br>WEAK

Table S2. The results of immunohistochemical evaluations for each normal (N) and tumor (T) specimen form melanoma samples (1 to 12) have been shown in the table. Results are displayed based on the following parameters:

- % of colored cells out of the total cells counted
- signal evaluation (cytosolic, nuclear)
- color intensity (weak, moderate, strong)

Tumor samples have been considered positive if the percentage of staining compared to the normal counterpart was >10% (due to RSK phosphorylation) or >=5% (due to MDM2 nuclear expression) and if the staining intensity was stronger in the tumor compared to the corresponding normal counterpart.

Samples 1,2,3,4,7,8,10 are positive for increased RSK phosphorylation and nuclear MDM2 overexpression.

Samples 5,6 are negative for increased RSK phosphorylation and nuclear MDM2 overexpression.

Samples 9,11,12 are positive for increased RSK phosphorylation and negative for nuclear MDM2 overexpression.
